# Inhibitory Effects of a Novel PPAR-*γ* Agonist MEKT1 on* Pomc* Expression/ACTH Secretion in AtT20 Cells

**DOI:** 10.1155/2018/5346272

**Published:** 2018-04-23

**Authors:** Rehana Parvin, Erika Noro, Akiko Saito-Hakoda, Hiroki Shimada, Susumu Suzuki, Kyoko Shimizu, Hiroyuki Miyachi, Atsushi Yokoyama, Akira Sugawara

**Affiliations:** ^1^Department of Molecular Endocrinology, Tohoku University Graduate School of Medicine, Sendai, Miyagi, Japan; ^2^Drug Discovery Initiative, The University of Tokyo, 7-3-1 Hongo, Bunkyo-ku, Tokyo, Japan

## Abstract

Although therapeutic effects of the peroxisome proliferator-activated receptor gamma (PPAR-*γ*) agonists rosiglitazone and pioglitazone against Cushing's disease have been reported, their effects are still controversial and inconsistent. We therefore examined the effects of a novel PPAR-*γ* agonist, MEKT1, on* Pomc* expression/ACTH secretion using murine corticotroph-derived AtT20 cells and compared its effects with those of rosiglitazone and pioglitazone. AtT20 cells were treated with either 1 nM~10 *μ*M MEKT1, rosiglitazone, or pioglitazone for 24 hours. Thereafter, their effects on proopiomelanocortin gene* (Pomc) *mRNA expression were studied by qPCR and the* Pomc* promoter (−703/+58) activity was demonstrated by luciferase assay.* Pomc* mRNA expression and promoter activity were significantly inhibited by MEKT1 at 10 *μ*M compared to rosiglitazone and pioglitazone. SiRNA-mediated PPAR-*γ* knockdown significantly abrogated MEKT1-mediated* Pomc* mRNA suppression. ACTH secretion from AtT20 cells was also significantly inhibited by MEKT1. Deletion/point mutant analyses of* Pomc* promoter indicated that the MEKT1-mediated suppression was mediated via NurRE, TpitRE, and NBRE at −404/−383, −316/−309, and −69/−63, respectively. Moreover, MEKT1 significantly suppressed* Nur77*,* Nurr1*, and* Tpit* mRNA expression. MEKT1 also was demonstrated to inhibit the protein-DNA interaction of Nur77/Nurr1-NurRE, Tpit-TpitRE, and Nur77-NBRE by ChIP assay. Taken together, it is suggested that MEKT1 could be a novel therapeutic medication for Cushing's disease.

## 1. Introduction

Peroxisome proliferator-activated receptor gamma (PPAR-*γ*) is a member of the nuclear receptor superfamily also known as ligand-inducible transcription factors [1]. Ligand binding with PPAR-*γ* receptor acts as a switch leading to the transcription complexes mediating repression or activation of transcription on specific target genes [2]. Moreover, PPAR-*γ* possesses beneficial pleotropic effects including anti-inflammatory and neuroprotective actions [3, 4] and antidiabetic [5–7], antineoplastic [8], and renoprotective effects [9]. PPAR-*γ* is expressed in normal human anterior pituitary as well as in adrenocorticotropic hormone- (ACTH-) secreting pituitary adenomas. Moreover, PPAR-*γ* expression was significantly higher in pituitary adenomas than normal pituitary tissues, and its expression in ACTH-secreting adenomas was significantly higher than any other types of pituitary adenomas [10–13]. ACTH, the product of proopiomelanocortin gene* (Pomc)*, is secreted from the corticotroph cells of the anterior pituitary.* Pomc* is exhibited in various tissues including pituitary (anterior and intermediate), hypothalamus, and skin [14].

The* Pomc* regulation is tissue-specific [15] and the regulatory mechanism of this gene has been elucidated in different tissues [16, 17] using different types of drugs. However, the PPAR-*γ*-mediated* Pomc* regulation mechanism has not yet been clarified in pituitary corticotroph cells. Moreover, preclinical studies conducted both* in vitro* and* in vivo* have provided the evidence of anticancer properties of particular PPAR-*γ* agonists, rosiglitazone and pioglitazone. Several studies demonstrating rosiglitazone and pioglitazone on* Pomc *suppression have been done [12, 18, 19], and an opposite effect of rosiglitazone was also shown by Kreutzer et al. [20]. Moreover, although rosiglitazone has been used as a therapeutic drug for the treatment of Cushing's disease due to its ability to reduce ACTH and corticosterone secretion in mouse corticotropic pituitary tumors, it has generally shown unsatisfactory results [21]. In addition, although previous studies have reported the therapeutic use of rosiglitazone and pioglitazone in Cushing's disease [11, 12, 22], there has been some controversy concerning these drugs [20, 23]. Since there have been few effective drugs for Cushing's disease, the discovery of novel drugs is very important to obtain a satisfactory treatment of Cushing's disease.

In this study, we examined the effects of a novel PPAR-*γ* agonist, MEKT1, on* Pomc *expression/ACTH secretion using murine pituitary corticotroph tumor-derived AtT20 cells and compared them with rosiglitazone and pioglitazone. We also examined the effects of MEKT1 on transcription factors Nur77, Nurr1, NeuroD1, and Tpit, which are known to activate* Pomc* transcription [24–26]. Our present study has indicated a possibility that MEKT1 may be a novel candidate for the therapeutic medication against Cushing's disease.

## 2. Materials and Methods

### 2.1. Reagents

MEKT1, a synthetic PPAR-*γ* agonist, was a gift from Okayama University. MEKT1 was dissolved in 100% DMSO at 10 mM and stored at −20°C. Rosiglitazone and pioglitazone hydrochloride were purchased from Sigma-Aldrich (St. Louis, MO) and Wako Pure Chemical Industries Ltd, Japan, respectively. 100% DMSO was used to dissolve rosiglitazone and pioglitazone hydrochloride at 10 mM and stored at −20°C. Before each experiment, these stored drugs were diluted with 100% DMSO to the desired concentration maintaining final concentration of DMSO at 0.1%.

### 2.2. Plasmids

Subcloned chimeric constructs which contained the rat* Pomc* genomic DNA and luciferase cDNA (pGL3-Basic, Promega, Madison, WI) were used for the studies of transient transfection: r*Pomc*-Luc (−703/+58-Luc: harboring the rat* Pomc* 5′-flanking region from −703 to +58 relative to the transcription start site upstream of the luciferase cDNA in pGL3-Basic), −429/+58-Luc, −379/+58-Luc, −359/+58-Luc, −293/+58-Luc, −169/+58-Luc, and +12/+58-Luc. Nur77/Nurr1 binding element in rPomc-Luc from 5′-TGATATTTACCTCC-3′ to 5′-cagcgcccACCTCC-3′ (rPomc-Luc-NurRE-Mut), Nur77 binding element in rPomc-Luc from 5′-AGGTCA-3′ to 5′-gtaTCA-3′ (rPomc-Luc-NBRE-Mut), and Tpit binding element in rPomc-Luc from 5′-TCACACC-3′ to 5′-gacCACC-3′ (rPomc-Luc-TpitRE-Mut). *β*-galactosidase control plasmid in pRSV (pRSV-*β*-gal) was purchased from Clontech (Mountain View, CA) and pcDNA3 expression plasmid from Invitrogen (Carsbad, CA). Murine Nur77, Tpit, and Nurr1 cDNA were cloned by PCR from AtT20 cells and were subcloned into the pcDNA3 expression vector (Invitrogen, Carlsbad, CA) to prepare Nur77-pcDNA3, Tpit-pcDNA3, and Nurr1-pcDNA3 [27, 28].

### 2.3. Cell Culture

AtT20 cells [28], obtained from the American Type Culture Collection (AtT20: CCL-89), were cultured with Dulbecco's modified Eagle medium (DMEM) added with 10% fetal bovine serum (FBS), 100 U/mL penicillin, and 100 *μ*g/mL streptomycin. Cells were cultured in a humidified incubator at 37°C with 5% CO_2_.

### 2.4. Proliferation Assay

The following procedure was outlined by Saito-Hakoda et al. [28]. Cell Counting Kit-8 (Dojindo, Kumamoto, Japan) was used for counting the cell numbers. Briefly, AtT20 cells (5 × 10^3^ cells/well) seeded in 96-well plates were incubated in 100 *μ*l regular media for few days. The cells were then refed with DMEM supplemented with 1% resin and charcoal-treated (stripped) FBS media containing appropriate concentrations of PPAR-*γ* agonist MEKT1. After 24-hour incubation, 10 *μ*l of assay reagent was added in each well and then the plate was incubated for 4 hours at 37°C, 5% CO_2_. The generation of the colored formazan product was measured optically by measuring the absorbance at 450 nm (reference 600 nm) using a microplate reader.

### 2.5. Measurement of Caspase 3 Activity

Caspase 3 activity was determined using a caspase 3/CPR32 Colorimetric Assay kit, according to the manufacturer's instructions (Biovision, Mountain View, CA 94043, USA). Briefly, the AtT20 cells were lysed in caspase 3 sample lysis buffer and incubate cells on ice for 10 minutes. The homogenates were then centrifuged at 10,000 ×g and 4°C for 1 min and the supernatant was collected for protein estimation. The cell lysates were then exposed to the DEVD substrate conjugate provided in the kit for 1 hour at 37°C. The sample was measured in an automatic microplate reader at an excitation of 400 nm.

### 2.6. RNA Isolation, cDNA Synthesis, and Quantitative Real-Time PCR

RNA isolation, cDNA synthesis, and quantitative real-time polymerase chain reaction (qPCR) were conducted as previously described [28, 29]. To confirm the amplification specificity, the PCR products from each primer pair with SYBR green were subjected to a melting curve analysis. For each sample, the expression of mRNA was normalized by dividing the expression of mouse GAPDH. The sequences of the primer sets are shown in Table 1.

### 2.7. Transient Transfection for Luciferase Assay

AtT20 cells were seeded to 60–70% confluence in regular medium in 24-multiwell plates and the cells were transfected (transiently) with 300 ng of each reporter plasmid and 100 or 150 ng of *β*-gal control plasmid. Transfection was carried out according to the manufacturer's instructions using Lipofectamine^(R)^ 2000 (Invitrogen). Each expression vector is of different concentrations (200 ng and 300 ng); 135 ng of reporter plasmid and 65 ng of *β*-gal control plasmid were also transfected with cells in overexpression experiments. Twenty-four hours after transfection, the medium was changed to DMEM added with 1% stripped FBS, and the cells were treated without or with MEKT1 (10 *μ*M) for the next 24 hours. Before luciferase assay, the cells were washed with 1x PBS and then the cell extracts were prepared using Glo Lysis Buffer (Promega) and *β*-galactosidase activity was also measured simultaneously. Data were normalized by *β*-galactosidase activity. We followed our previously published protocol [29].

### 2.8. Small Interfering RNA

Small interfering RNAs (siRNAs) for PPAR-*γ* (NM_011146_stealth_342) [30] and negative control siRNA (ID: 1022076) were obtained from Qiagen (Hilden, Germany). AtT20 cells were cultured to 50% confluence in 24-multiwell plates transiently transfected with 10 pmol siRNAs using Lipofectamine^(R)^ 2000 (Invitrogen) for 48 hours according to the manufacturer's instructions. The cells were then incubated either without or with 10 *μ*M MEKT1 for 24 hours and then used for quantitative RT-PCR. Reporter plasmids were transfected with the cells and then incubated either without or with MEKT1 at 10 *μ*M for 24 hours and these cells were used for luciferase assay.

### 2.9. Enzyme Immunoassay (EIA)

EIA was performed for measuring of ACTH concentration. AtT20 cells were cultured to 60% confluence in regular medium in 24-multiwell plates and then incubated either without or with at appropriate concentrations of MEKT1, rosiglitazone, and pioglitazone hydrochloride in DMEM added with 1% stripped FBS for 24 hours. The ACTH concentration in the supernatants was measured by an ACTH (rat. mouse) EIA kit (Phoenix Pharmaceuticals, Burlingame, CA). Data were normalized by the total protein in each well.

### 2.10. Western Blot Analyses

AtT20 cells were grown to 70% confluence in regular medium in 6 cm dishes, and they were incubated in the presence rosiglitazone, pioglitazone, and MEKT1 (time dependently) or in the presence of 100% DMSO in DMEM supplemented with 1% stripped FBS media for 24 hours. The cells were then harvested and lysed with TNE buffer (20 mmol/L Tris-HCl, 137 mmol/L NaCl, 2 mmol/L EDTA, 1% NP-40, Protease Inhibitor Cocktail Set III (Calbiochem), pH 7.9). Thereafter, 20 *μ*g of extracted protein was electrophoresed on a SDS-polyacrylamide gel and transferred onto PVDF membrane. For the detection of NURR1, Nur77, and TBX19 (Tpit) protein the membrane was blocked with 1% BSA for 30 minutes and probed with the primary antibody for Nur77/Nurr1 antibody (SC-990, Santa Cruz Biotechnology); anti TBX19 antibody (GTX77878, GeneTex); Nur77 (ab13851, Abcam) diluted at 1 : 1000 with 1% BSA, for overnight at 4°C, and was thereafter incubated with anti-rabbit IgG, horseradish peroxidase (HRP) linked whole antibody from donkey (NA934V, GE Healthcare Life Sciences, Pittsburgh, PA) (1 : 5000) for 1 hour at room temperature. For the detection of actin, the membrane was blocked with 1% BSA for 30 minutes at room temperature and probed with the primary antibody for actin (sc-1616, Santa Cruz Biotechnology) (diluted at 1 : 500) for overnight at 4°C and was thereafter incubated with anti-goat IgG, HRP preabsorbed from donkey (ab97120) (1 : 5000) for 1 hour at room temperature. Thereafter, the membranes were washed and were visualized using ECL (Bio-Rad). Densitometric analyses of the membranes were performed using Image J.

### 2.11. Chromatin Immunoprecipitation (ChIP) Assay

ChIP assay was performed using anti-Nur77/Nurr1 antibody (SC-990, Santa Cruz Biotechnology); anti TBX19 antibody (GTX77878, GeneTex); normal rabbit IgG (SC-2027, Santa Cruz Biotechnology); and NurRE (Nur response element), TpitRE (Tpit response element), and NBRE (Nur77 response element) region containing primers of mouse* Pomc* promoter. ChIP assay was conducted as described previously [29]. DNA fragments were treated with Proteinase K (Wako, Osaka, Japan) and Qiagen DNA Extraction kit was used for purification of DNA. Immunoprecipitated DNA was analyzed by qPCR when KAPA SYBR FAST Universal 2x qPCR Master Mix (KAPA Biosystems) reagent was used for qPCR. Data were represented as enrichment of the immunoprecipitated DNA compared to 1% input DNA. NurRE, TpitRE, and NBRE region specific primer pairs of mouse* Pomc* promoter were designed to amplify by qPCR. The sequences of the primer sets are shown in Table 2.

### 2.12. Statistical Analyses

Data are displayed as means ± standard errors of means (SEM). Statistical analysis was performed with one way ANOVA followed by Tukey's post hoc test among the groups and Paired Sample *t* test between the groups. *P* value < 0.05 was considered as statistically significant. Statistical details are found in the Figures and Figure legends.

## 3. Results

### 3.1. Effects of PPAR-*γ* Agonists Rosiglitazone, Pioglitazone, and MEKT1 on mRNA Expression/Promoter Activity of* Pomc*

**Figure 1:**
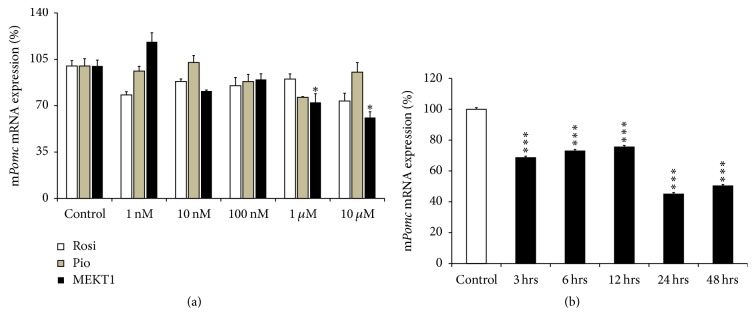
Effects of MEKT1, rosiglitazone, and pioglitazone on mRNA expression of* Pomc* in AtT20 cells. (a) Effects of MEKT1, rosiglitazone, and pioglitazone on mRNA expression of* Pomc* dose-dependently. AtT20 cells were treated with MEKT1, rosiglitazone (Rosi), and pioglitazone (Pio) (1 nM, 10 nM, 100 nM, 1 *μ*M, or 10 *μ*M) or 0.1% DMSO (vehicle control) for 24 hours. ^*∗*^*P* < 0.05 versus control. (b) Effect of MEKT1 on* Pomc* mRNA expression time dependently. AtT20 cells were treated with 10 *μ*M MEKT1 for 1 hour, 3 hours, 6 hours, 12 hours, or 24 hours. Vehicle control, 0.1% DMSO. Data are expressed as percentages (100%) of control. Each point indicates mean ± SEM (*n* = 4). ^*∗∗∗*^*P* < 0.001 versus control.

We first analyzed the effects of rosiglitazone, pioglitazone, and MEKT1 on mRNA expression of* Pomc* at various concentrations in AtT20 cells. After treatment of the cells with various concentrations (1 nM, 10 nM, 100 nM, 1 *μ*M, and 10 *μ*M) of rosiglitazone, pioglitazone, and MEKT1,* Pomc *mRNA was significantly decreased at 1 *μ*M and 10 *μ*M of MEKT1, but no significant suppressive effects were observed when rosiglitazone and pioglitazone were added (Figure 1(a)). Next we examined the MEKT1-mediated effect on* Pomc* mRNA expression using different durations of incubation in the cells. After treatment of the cells with MEKT1 (10 *μ*M) for 3 hours, 6 hours, 9 hours, 24 hours, or 48 hours, the* Pomc *mRNA expression was significantly decreased from 3 hours to 48 hours in a time dependent manner (Figure 1(b)). These results indicated that MEKT1 decreased mRNA expression of* Pomc* both dose- and time dependently. In contrast, MEKT1 dose-dependently increased PPAR-*γ* mRNA expression (Figure S1).

**Figure 2:**
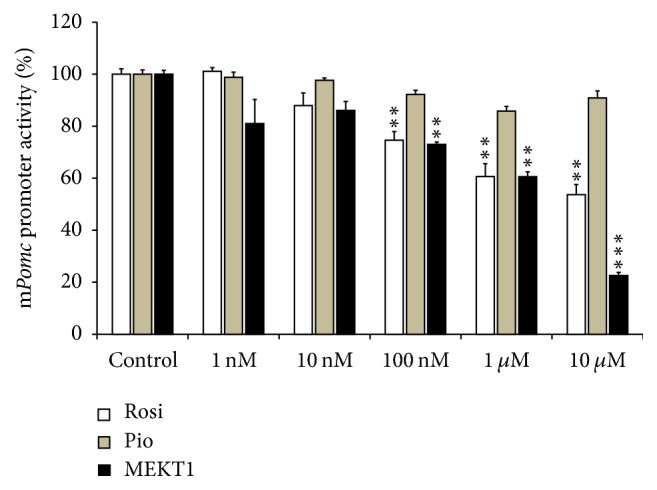
Effects of MEKT1, rosiglitazone, and pioglitazone on* Pomc* promoter activity in AtT20 cells. AtT20 cells transiently transfected with 300 ng full length r*Pomc*-Luc (−703/+58-luc) and 100 ng pRSV-*β*-gal were treated with MEKT1, rosiglitazone (Rosi), and pioglitazone (Pio) (1 nM, 10 nM, 100 nM, 1 *μ*M, or 10 *μ*M) or 0.1% DMSO (vehicle control) for 24 hours. Data are expressed as percentages (100%) of control. Each point represents mean ± SEM (*n* = 4). ^*∗∗*^*P* < 0.01, ^*∗∗∗*^*P* < 0.001 versus control.

We next examined the effects of rosiglitazone, pioglitazone, and MEKT1 on the promoter activity of* Pomc* using AtT20 cells. In this experiment, the full length (−703/+58)* Pomc* promoter was used with different concentrations of rosiglitazone, pioglitazone, and MEKT1. As shown in Figure 2, MEKT1 significantly suppressed the promoter activity of* Pomc* dose-dependently, whereas pioglitazone had no suppressive effect. Though rosiglitazone had a suppressive effect on* Pomc* promoter activity, the effect was less strong than that of MEKT1. These results indicated that MEKT1-mediated negative regulation of* Pomc* transcription is most effective than rosiglitazone and pioglitazone.

### 3.2. Effects of PPAR-*γ* Agonists MEKT1, Rosiglitazone, and Pioglitazone on ACTH Secretion

**Figure 3:**
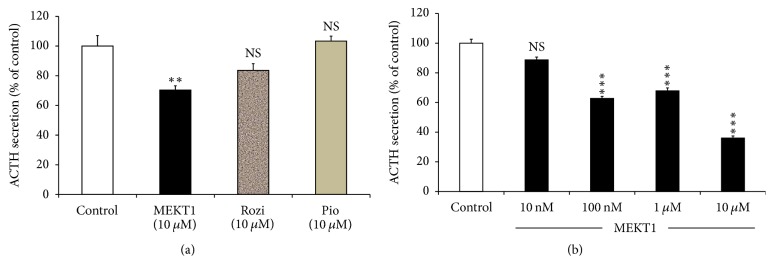
Effects of MEKT1, rosiglitazone, and pioglitazone on ACTH secretion from AtT20 cells. (a) Effects of MEKT1, rosiglitazone, and pioglitazone on ACTH secretion into the media from AtT20 cells. AtT20 cells were treated with MEKT1, rosiglitazone (Rosi), and pioglitazone (Pio) (10 *μ*M) or DMSO (0.1%) as a control. After 24-hour incubation of the cells, the ACTH secreted to the media was determined by EIA. (b) Dose-dependent effects of MEKT1 on ACTH secretion into the media from AtT20 cells. AtT20 cells were treated with MEKT1 (10 nM, 100 nM, 1 *μ*M, or 10 *μ*M) or 0.1% DMSO (vehicle control) for 24 hours. After 24-hour incubation of the cells, the ACTH secreted to the media was determined by EIA. Data are expressed as percentages (100%) of control. Each point represents mean ± SEM (*n* = 4). NS means “not significant.” ^*∗∗∗*^*P* < 0.001, ^*∗∗*^*P* < 0.01 versus control.

We identified the effects of PPAR-*γ* agonists MEKT1, rosiglitazone, and pioglitazone at 10 *μ*M on ACTH secretion of AtT20 cells in the supernatant and observed that only MEKT1 significantly suppressed ACTH secretion (Figure 3(a)), whereas there was no significant effect of rosiglitazone and pioglitazone on it. Due to the significant suppression of MEKT1 on ACTH secretion, we then examined the dose-dependent effects of MEKT1 on ACTH secretion. In this experiment, AtT20 cells were treated with different concentrations of MEKT1 (10 nM, 100 nM, 1 *μ*M, and 10 *μ*M) in Figure 3(b). MEKT1 significantly suppressed ACTH secretion from 100 nM to 10 *μ*M.

### 3.3. Effects of MEKT1 on AtT20 Cell Proliferation and Apoptosis in AtT20 Cells

**Figure 4:**
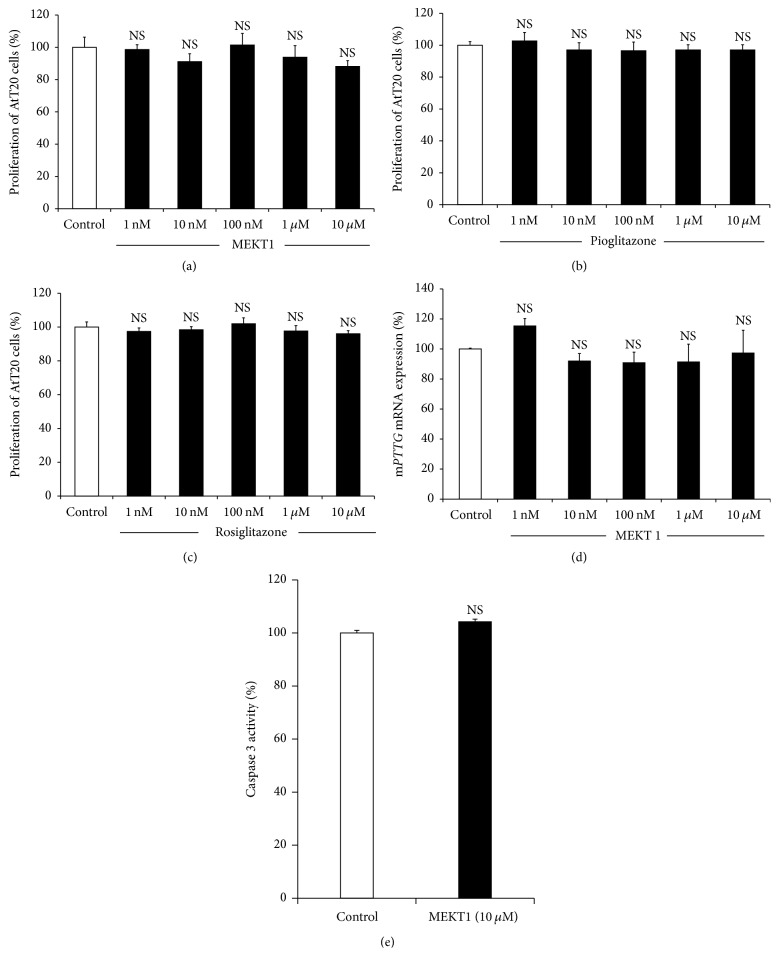
MEKT1-mediated effect on AtT20 cell proliferation and apoptosis. (a) AtT20 cells were incubated for 96 hours either in the presence of MEKT1 (1 nM, 10 nM, 100 nM, 1 *μ*M, or 10 *μ*M) or DMSO (0.1%) as a control for 24 hours before assay. (b) AtT20 cells were incubated for 96 hours either in the presence of pioglitazone (1 nM, 10 nM, 100 nM, 1 *μ*M, or 10 *μ*M) or DMSO (0.1%) as a control for 24 hours before assay. (c) AtT20 cells were incubated for 96 hours either in the presence of rosiglitazone (1 nM, 10 nM, 100 nM, 1 *μ*M, or 10 *μ*M) or DMSO (0.1%) as a control for 24 hours before assay. Data are expressed as percentages (100%) of control. (d) Effects of MEKT1 on mRNA expression of m*Pttg* dose-dependently. AtT20 cells were treated with MEKT1 (1 nM, 10 nM, 100 nM, 1 *μ*M, or 10 *μ*M) or 0.1% DMSO (vehicle control) for 24 hours. (e) Effects on MEKT1 (10 *μ*M) on AtT20 cell apoptosis. Each point indicates mean ± SEM (*n* = 4). NS stands for “not significant.”

We examined the effects of MEKT1 on proliferation of AtT20 cells using a WST-8 assay after incubation with various concentrations from 1 nM to 10 *μ*M for 96 hours. MEKT1 did not exert any inhibitory effect on the proliferation of AtT20 cells from 1 nM to 10 *μ*M (Figure 4(a)). Although treatment of rosiglitazone and pioglitazone for 24 hours did not exhibit (Figures 4(b) and 4(c)) inhibitory effect on AtT20 cell proliferation, treatment of rosiglitazone for 48 hours inhibited the cell proliferation of corticotroph tumor cells [12]. These data indicated that the MEKT1 had no toxic effect on the AtT20 cells at concentrations of 10 *μ*M. Next we examined the effect of MEKT1 on AtT20 cell apoptosis by caspase-3 assay and observed no apoptotic activity of MEKT1 in Figure 4(e) on AtT20 cells. Moreover, we also demonstrated the effect of MEKT1 on the mRNA expression of the proliferative marker, pituitary tumor transforming gene* (Pttg)* in Figure 4(d), and observed no effect of MEKT1 on* Pttg*.

### 3.4. The Involvement of PPAR-*γ* in the MEKT1-Mediated Suppression of mRNA Expression/Promoter Activity of* Pomc*

**Figure 5:**
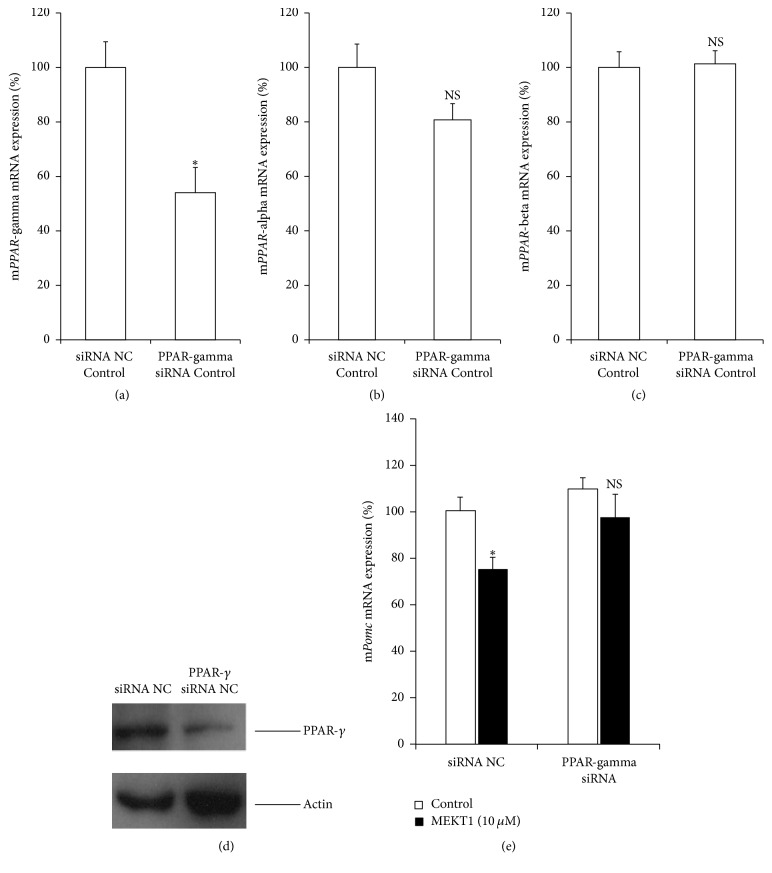
Involvement of PPAR-*γ* in the MEKT1 effects on* Pomc* mRNA expression. Effects of PPAR-*γ* knockdown by its siRNA on (a)* PPAR-γ* mRNA expression, (b)* PPAR-α *mRNA expression, and (c)* PPAR-β* mRNA expression. AtT20 cells transiently transfected with siRNA (negative control; NC or PPAR-*γ*) for 48 hours were incubated with 0.1% DMSO (control) for 24 hours. Results are expressed as percentages of each control. Each point represents mean ± SEM (*n* = 4). ^*∗*^*P* < 0.05 versus basal negative control siRNA. NS stands for “not significant.” (d) Effects of PPAR-*γ* knockdown by its siRNA on the PPAR-*γ* protein expression. (e) Effects of PPAR-*γ* knockdown by its siRNA on the* Pomc* mRNA expression. AtT20 cells transiently transfected with siRNA (negative control; NC or PPAR-*γ*) for 48 hours were incubated in the presence of either MEKT1 (10 *μ*M) or 0.1% DMSO (control) for 24 hours, respectively. Data are expressed as percentages (100%) of control. Each point represents mean ± SEM (*n* = 4). NS stands for “not significant.” ^*∗*^*P* < 0.05 versus negative control siRNA at 10 *μ*M MEKT1.

We examined the involvement of PPAR-*γ* in the MEKT1-mediated suppression of mRNA expression and promoter activity of* Pomc* by knocking down its small interfering RNA (siRNA). The decrease of endogenous* PPAR*-*γ* mRNA expression by its siRNA was confirmed by qPCR, as shown in Figure 5(a). Moreover, endogenous* PPAR*-*α* and* PPAR*-*β* mRNA expression were not observed in Figures 5(b) and 5(c), respectively. The decrease of PPAR-*γ* protein expression by its siRNA was confirmed by western blot analysis, as shown in Figure 5(d). PPAR-*γ* siRNA significantly abrogated the suppression of* Pomc* mRNA expression by MEKT1 (Figure 5(e)). Moreover, PPAR-*γ* siRNA significantly abrogated the MEKT1-mediated suppression of* Pomc* promoter activity (Figure S2). These results indicate that the negative regulation of* Pomc* expression by MEKT1 is most likely mediated via PPAR-*γ*.

### 3.5. Effects of MEKT1 on the* Pomc* Promoter Deletion Mutants, and the Involvement of NurRE, TpitRE, and NBRE in the MEKT1-Mediated Suppression of* Pomc* Promoter Activity

**Figure 6:**
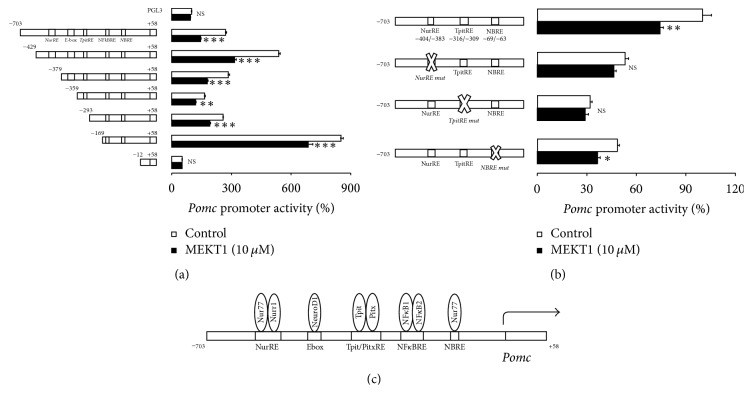
Effects of MEKT1 on* Pomc* promoter deletion mutants and role of NurRE, TpitRE, and NBRE on MEKT1-mediated effect on* Pomc* promoter activity in AtT20 cells. (a) MEKT1-mediated effect on* Pomc* promoter deletion mutants. AtT20 cells transiently transfected with 300 ng rPomc-Luc (−703/+58-luc) or each deletion mutant reporter plasmid (−429/+58-Luc, −379/+58-Luc, −359/+58-Luc, −293/+58, −169/+58, and +12/+58) and 100 ng pRSV-*β*-gal were incubated in the presence (10 *μ*M) or absence of MEKT1 for 24 hours before the luciferase assay. Data are expressed as percentages of each control (100% in pGL3-Basic). (b) MEKT1-mediated effect on* Pomc* promoter activity using NurRE mut, TpitRE mut, and NBRE mut. AtT20 cells transiently transfected with 300 ng r*Pomc*-Luc (−703/+58-luc) or NurRE mut (r*Pomc*-Luc- NurRE -Mut), TpitRE mutant (r*Pomc*-Luc- TpitRE -Mut), NBRE mutant of* Pomc* promoter (r*Pomc*-Luc- NBRE -Mut) of* Pomc* full length promoter and 150 ng pRSV-*β*-gal were incubated in the presence (10 *μ*M) or absence of MEKT1 for 24 hours before the luciferase assay. Data are expressed as percentages of each control (100% in r*Pomc*-Luc). Data represent mean ± SEM (*n* = 4). NS denotes “not significant.” ^*∗*^*P* < 0.05, ^*∗∗*^*P* < 0.01, and ^*∗∗∗*^*P* < 0.001 versus control. (c) Graphical representation of responsive elements on the promoter of* Pomc* and transcription factors which bind to the responsive elements of* Pomc* promoter.

We next examined the molecular mechanisms of* Pomc* transcription regulation by MEKT1. Therefore, we analyzed the promoter activity of* Pomc* 5′-flanking region deletion mutants series and it was observed that transcription suppression of* Pomc* promoter activity by MEKT1 was found in constructs from −703/+58 to −169/+58, but not in −12/+58 (Figure 6(a)). The luciferase activity of pGL3-Basic vector was unaffected by MEKT1 (Figure 6(a)).* Pomc* promoter constructs from −703/+58 to −169/+58 contained the NurRE, TpitRE, and NBRE, whereas the −12/+58 construct contained no responsive elements of* Pomc* promoter. It is plausible that NurRE, TpitRE, and NBRE probably exert an influential role in transcription suppression of* Pomc*, which occurred by MEKT1. To confirm the role of NurRE, TpitRE, and NBRE in the suppression of* Pomc* transcription, we further demonstrated the impact of MEKT1 on the NurRE, TpitRE, and NBRE mutants (Figure 6(b)). As shown in Figure 6(b), NurRE and TpitRE mutants completely abrogated the MEKT1-mediated repression of* Pomc* promoter activity, while NBRE mutant partially abrogated the MEKT1-mediated repression of* Pomc* promoter activity. Therefore, NurRE and TpitRE are important for the transcription suppression of* Pomc* promoter activity, which was mediated by MEKT1. These data suggest that NurRE and TpitRE play a prominent role in the MEKT1-mediated negative regulation of* Pomc *transcription.  Figure 6(c) represents the structure of the rat* Pomc *promoter. Since Nur77/Nurr1 [31] is known to bind to NurRE, and Tpit is known to bind to TpitRE [32], Nur77/Nurr1 and Tpit may be involved in the MEKT1-mediated suppression of* Pomc* promoter activity.

### 3.6. Effects of MEKT1 on mRNA Expression of* Nur77*,* Nurr1*,* NeuroD1*,* Tpit*,* Pitx*,* NFkB1*, and* NFkB2*

**Figure 7:**
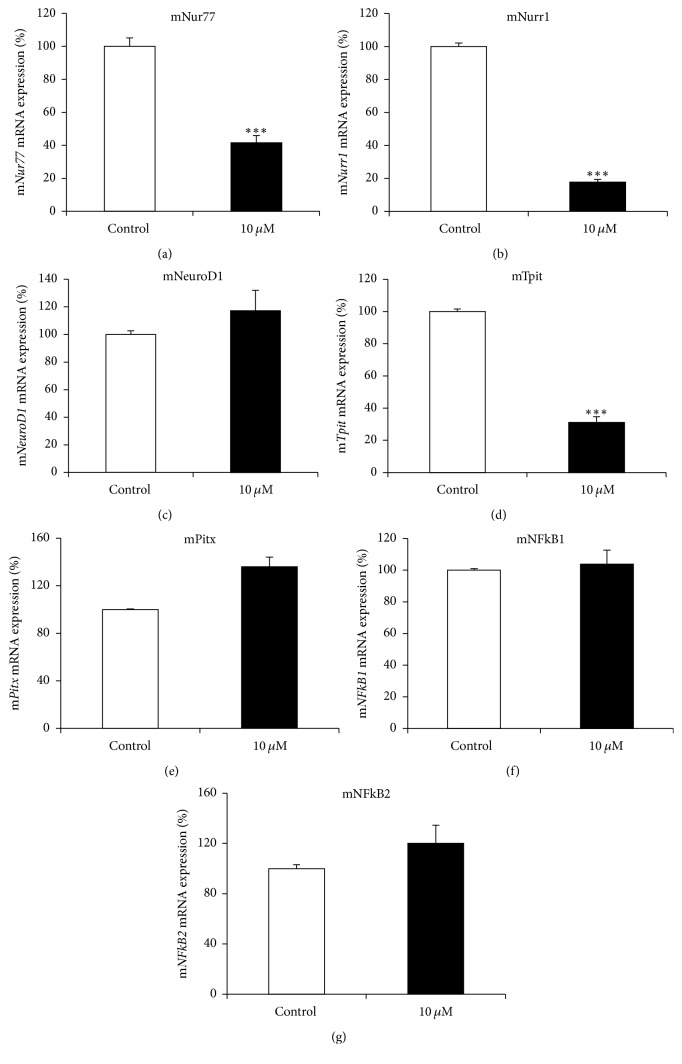
MEKT1-mediated effects on the mRNA expression of mouse* Nur77*,* Nurr1*,* NeuroD1*,* Tpit*,* Pitx*,* NFkB1*, and* NFkB2 *in AtT20 cells. AtT20 cells treated with MEKT1 (10 *μ*M) or 0.1% DMSO (vehicle control) for 24 hours. (a)* Nur77* mRNA expression, (b)* Nurr1* mRNA expression, (c)* NeuroD1* mRNA expression, (d)* Tpit *mRNA expression, (e)* Pitx* mRNA expression, (f)* NFκB1 *mRNA expression, and (g)* NFκB2 *mRNA expression. Data are expressed as percentages (100%) of control. Data represent mean ± SEM (*n* = 4). ^*∗∗∗*^*P* < 0.001 versus control.

**Figure 8:**
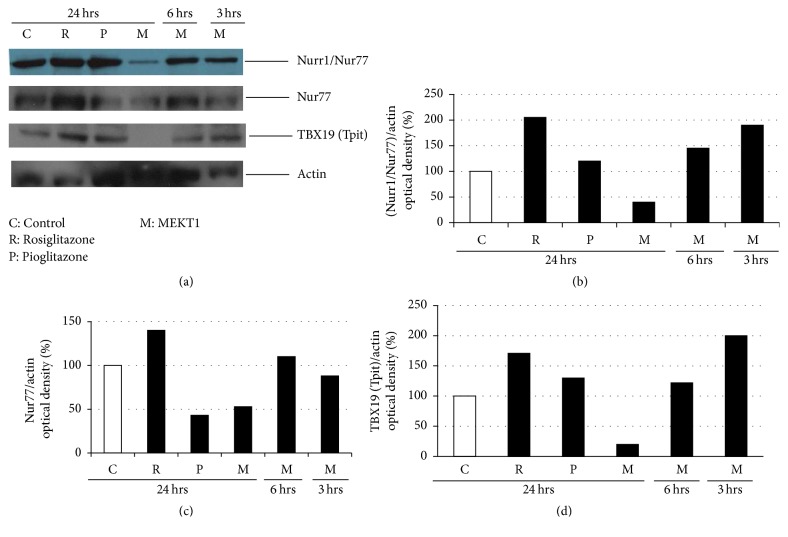
Effects of MEKT1 (time dependently), rosiglitazone, and pioglitazone on Nurr1, Nur77, and Tpit protein expression. (a) AtT20 cells treated with MEKT1 (M) at 10 *μ*M for 24 hours, 6 hours, and 3 hours, rosiglitazone (R) at 10 *μ*M for 24 hours, and pioglitazone (P) at 10 *μ*M for 24 hours, or 0.1% DMSO as control (C) for 24 hours. Optical density (OD) of Nurr1/Nur77 was shown in figure (b), Nur77 in figure (c), and TBX19 (Tpit) in figure (d). OD of Nurr1/Nur77, Nur77, and TBX19 (Tpit) were normalized by OD of actin. Results are expressed as percentages of control (100%).

We next examined the effect of MEKT1 on mouse* Nur77*,* Nurr1*,* NeuroD1*,* Tpit*,* Pitx*,* NFkB1*, and* NFkB2* mRNA expression in AtT20 cells. As shown in Figures 7(a), 7(b), and 7(d), MEKT1 decreased mRNA expression of* Nur77*,* Nurr1*, and* Tpit *at the concentration of 10 *μ*M but not that of* NeuroD1*,* Pitx*,* NFkB1*, and* NFkB2* (Figures 7(c), 7(e), 7(f), and 7(g)). We also demonstrated the effect of MEKT1 on Nurr1, Nur77, and Tpit protein expression (Figure 8) in AtT20 cells. As shown in Figures 7(a), 7(b), and 7(d), results suggest that the MEKT1-mediated suppression of* Pomc* transcription probably was implicated via the suppression of* Nur77*,* Nurr1*, and* Tpit* mRNA expression which was confirmed by suppression of Nurr1, Nur77, and Tpit protein expression in Figure 8. We next examined the effects of MEKT1 at several concentrations (1 nM, 10 nM, 100 nM, 1 *μ*M, and 10 *μ*M) on mouse* Nur77*,* Nurr1*, and* Tpit* mRNA expression as shown in Figures S3A, S3B, and S3C, and observed its dose-dependent effects.

### 3.7. Effects of Nur77, Tpit, and Nurr1 Overexpression on the MEKT1-Mediated Suppression of mRNA Expression/Promoter Activity of* Pomc*

**Figure 9:**
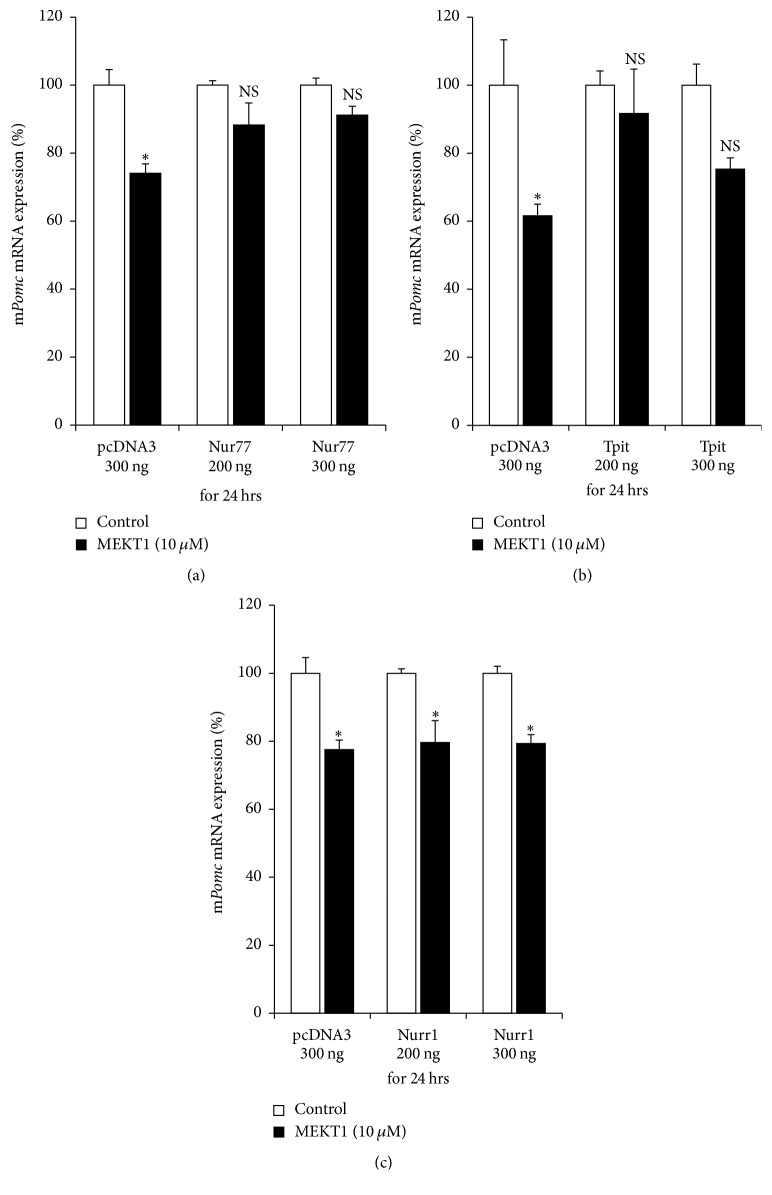
Effects of Nur77, Tpit, and Nurr1 overexpression on MEKT1-mediated effect of* Pomc* mRNA expression. (a) Nur77 overexpression effect on the MEKT1-mediated suppression of* Pomc* mRNA expression in AtT20 cells. AtT20 cells transiently transfected with pcDNA3 and Nur77 overexpression plasmid were incubated either in the presence of MEKT1 at 10 *μ*M or DMSO at 0.1% (control) for 24 hours. (b) Tpit overexpression effect on the MEKT1-mediated suppression of* Pomc* mRNA expression. AtT20 cells transiently transfected with pcDNA3 and Tpit overexpression plasmid were incubated either in the presence of MEKT1 at 10 *μ*M or DMSO at 0.1% (control) for 24 hours. (c) Nurr1 overexpression on the MEKT1-mediated suppression of* Pomc* mRNA expression. AtT20 cells transiently transfected with pcDNA3 and Nurr1 overexpression plasmid were incubated either in the presence of MEKT1 at 10 *μ*M or DMSO at 0.1% (control) for 24 hours. Each overexpression plasmid volume was maintained to 300 ng adding pcDNA3 empty vector. Results are expressed as percentages (100%) of control. Data represent mean ± SEM (*n* = 4). NS stands for “not significant.” ^*∗*^*P* < 0.05, versus control.

We next performed the overexpression of Nur77, Tpit, and Nurr1 to examine the role of Nur77, Tpit, and Nurr1 in the MEKT1-mediated suppression of mRNA expression and promoter activity of* Pomc*. As shown in Figures 9(a) and 9(b), overexpression of Nur77 and Tpit recovered the MEKT1-mediated repression of* Pomc* mRNA expression, when respective control plasmid (pcDNA3) could not recover. As shown in Figures S4A and S4B, overexpression of Nur77 and Tpit recovered the MEKT1-mediated suppression of* Pomc* promoter activity, while respective control plasmid (pcDNA3) could not. Overexpression of Nurr1 did not recover the MEKT1-mediated suppression of* Pomc* mRNA expression (Figure 9(c)) and* Pomc* promoter activity (Figure S4C). These data suggest the involvement of Nur77 and Tpit transcription factor in the MEKT1-mediated suppression of* Pomc*.

### 3.8. Effects of MEKT1 on the Interaction between Nur77/Nurr1 and NurRE, Tpit and TpitRE, and Nur77 and NBRE on the* Pomc* Promoter

**Figure 10:**
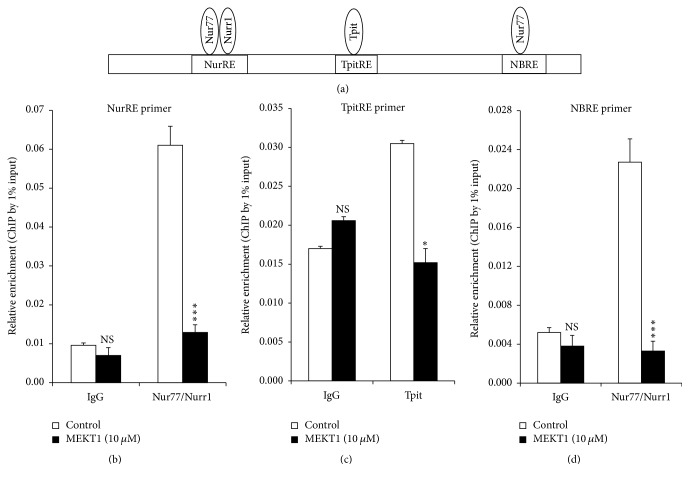
Effects of MEKT1 on the interaction between Nur77/Nurr1 and NurRE, Tpit and TpitRE, and Nur77 and NBRE on* Pomc* promoter (a) in AtT20 cells. Effects of MEKT1 on the interaction between Nur77/Nurr1 and NurRE (b), Tpit and TpitRE (c), and Nur77 and NBRE (d) on* Pomc* promoter examined by ChIP assay using NurRE, TpitRE, and NBRE primer. ChIP assay was carried out using digested chromatin extracted from the cells cultured in the presence of either 10 *μ*M MEKT1 or 0.1% DMSO (control) for 24 hours. Chromatin fragments were immunoprecipitated either by normal rabbit IgG (negative control), anti-Nur77/Nurr1 antibody, or anti-Tpit (anti TBX 19) antibody. Purified DNA was analyzed by qPCR using primers specific for NurRE, TpitRE, and NBRE containing sequence on* Pomc* promoter. The primer product sizes of NurRE, TpitRE, and NBRE were 211 bp, 146 bp, and 102 bp, respectively. Immunoprecipitated DNA was amplified by qPCR and then normalized to the values obtained after amplification of immunoprecipitated 1% input DNA. Data represent mean ± SEM (*n* = 3). NS means “not significant.” ^*∗*^*P* < 0.05, and ^*∗∗∗*^*P* < 0.001 significantly different from the level of control group.

Since Tpit, Nur77/Nurr1, and Nur77 transcription factors are known to bind to NurRE, TpitRE, and NBRE, respectively, on the* Pomc* promoter [25, 31, 33], we next analyzed the influence of MEKT1 on the interaction between Nur77/Nurr1 and NurRE, Tpit and TpitRE, and Nur77 and NBRE on its promoter of by ChIP assay using primers comprising NurRE, TpitRE, and NBRE (Figure 10(a)). As shown in Figures 10(b)–10(d), MEKT1 significantly suppressed the interaction between Nur77/Nurr1 and NurRE, Tpit and TpitRE, and Nur77 and NBRE on the* Pomc* promoter (24 hours), while it did not affect their interaction when IgG control was used. These data suggest that MEKT1 specifically inhibited their protein-DNA interactions.

## 4. Discussion

More than a decade ago, PPAR-*γ* agonist has been discovered as a new therapeutic medication for Cushing's disease [11, 12, 22]. Furthermore, it was reported that the PPAR-*γ* agonists rosiglitazone and pioglitazone target pituitary tumors* in vitro* and* in vivo* in Cushing's disease [11, 12, 22, 34, 35]. In the present study, we found that MEKT1 significantly suppressed the* Pomc *mRNA expression (Figure 1) and* Pomc* promoter activity after 24 hours of treatment at 10 *μ*M (Figure 2). In addition, comparing the effects of the three PPAR-*γ* agonists MEKT1, rosiglitazone, and pioglitazone on ACTH secretion in AtT20 cells (Figure 3), it was clearly shown that MEKT1 more significantly suppressed the* Pomc* expression and the ACTH secretion than rosiglitazone and pioglitazone. However, Heaney et al. [12] showed that rosiglitazone can suppress* Pomc* promoter activity significantly after 48 hours. Taken together it was shown that MEKT1 is more effective than rosiglitazone and pioglitazone in suppressing* Pomc *expression. It was also determined that the potency of MEKT1 was much greater than rosiglitazone in HEK293 cells [36]. We also confirmed using PPAR-*γ* siRNA that the MEKT1-mediated effect on* Pomc* expression was mediated via PPAR-*γ*.

However, the negative regulatory mechanism of the* Pomc* transcription by PPAR-*γ* is still unknown. Therefore, we also attempted to elucidate the molecular mechanism of the MEKT1-mediated suppression of* Pomc* transcription regulation. To clarify the molecular mechanism, we firstly demonstrated the effects of MEKT1 on* Pomc* promoter deletion mutants of different lengths −703/+58 (full length), −429/+58, −379/+58, −359/+58, −169/+58, and −12/+58, which possess different responsive elements (Figure 6). Moreover, although we also examined the effects of MEKT1 on the promoter activity of* Pomc* using −62/+12 deletion mutants (data not shown), we did not observe any MEKT1-mediated suppression of* Pomc* promoter activity, most likely due to the lack of NurRE/TpitRE/NBRE elements. This experiment showed the importance of the responsive elements NurRE, TpitRE, and NBRE in the MEKT1-mediated suppression of* Pomc* promoter activity. In this study, we first demonstrated the molecular mechanism of the PPAR-*γ*-mediated negative regulation of* Pomc*.

Moreover, it is already established that Nur77/Nurr1, NeuroD1, Tpit, Pitx, NF*κ*B1, and NF*κ*B2 are important transcription factors for* Pomc* expression [25, 26, 33, 37–40]. Therefore, we next examined the effects of MEKT1 on these transcription factors. Although* Nur77*,* Nurr1*, and* Tpit* mRNA expression and Nur77, Nurr1, and Tpit protein expression were significantly suppressed by MEKT1 in AtT20 cells,* NeuroD1*,* Pitx*,* NFκB1*, and* NFκB2* mRNA expression were not affected by MEKT1 (Figures 7(a)–7(g), and 8). Therefore, it was predicted that the MEKT1-mediated suppression of* Nur77*,* Nurr1*, and* Tpit* mRNA expression was possibly implicated in the MEKT1-mediated suppression of* Pomc* transcription and Pomc translation. Since several transcription factor binding sites are present on the* Pomc* promoter [40], simultaneous interactions among these regulatory elements are needed for* Pomc* transcription in the pituitary [41]. The proximal binding sequence termed NBRE (−69/−63) is known to be bound by the Nur77 monomer [31, 33] and the distal NurRE, composed of two inverted NBRE related sites (−404/−397 and −390/−383) is recognized to be bound by the Nur77/Nurr1 heterodimer or Nur77 homodimer. Compared to the proximal NBRE, distal NurRE responds to Nur77 in much stronger fashion [31, 33, 40].

In addition, NF-*κ*B RE (−151/−142) [39, 40], Tpit/PitxRE (−316/−309 and −302/−297) [32, 40], and E-box (−377/−370) [29, 38, 40] are known to be involved in regulation of* Pomc*. Based on these data, we again examined the transcriptional activity of the site directed mutation of NurRE (NurRE mut), TpitRE (TpitRE mut), and NBRE (NBRE mut). NurRE and TpitRE mutants completely abolished the MEKT1-mediated suppressive effect of the* Pomc* promoter activity. Therefore, it can be assumed that NurRE and TpitRE are the most important responsive elements for the MEKT1-mediated suppression of the* Pomc* promoter activity. Although NBRE mutant partially abolished the MEKT1-mediated suppressive effect due to the weak interaction of Nur77 monomer and NBRE [31], it is still noteworthy that MEKT1 significantly inhibited the interaction between Nur77 and NBRE on* Pomc* promoter in the ChIP assay (Figure 10). To verify the importance of transcriptional factors Nur77, Nurr1, and Tpit, we also performed an overexpression experiment and observed that the MEKT1-mediated suppression of* Pomc* promoter activity was attenuated by the Nur77 and Tpit overexpression (Figure 9).

## 5. Conclusion

**Figure 11:**
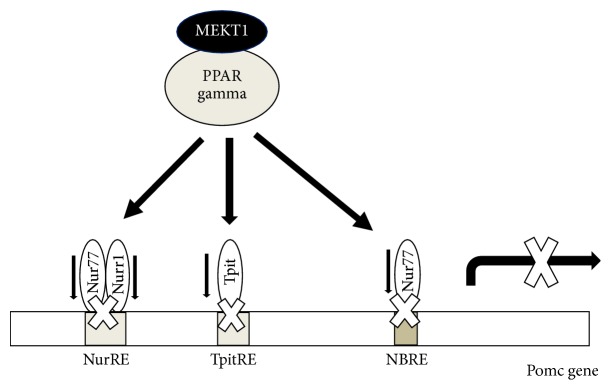
Involvement of Nur77, Nurr1, and Tpit transcription factors in the MEKT1 (PPAR-*γ* agonist)-mediated suppression of* Pomc*.

We can conclude as shown in Figure 11 that Nur77/Nurr1 heterodimer binding element NurRE (−383/−404), Tpit responsive element TpitRE (−309/−316), and Nur77 monomer responsive element NBRE (−63/−69) play important roles in* Pomc *expression [32, 33, 40]. When PPAR-*γ* agonist MEKT1 is added, it decreases* Nur77*,* Nurr1*, and* Tpit* mRNA expression and then probably inhibits the interactions between Nur77/Nurr1 heterodimer and NurRE, Tpit and TpitRE, and Nur77 monomer and NBRE (Figure 11), resulting the suppression of* Pomc* expression. Therefore, it can be concluded that Nur77, Nurr1, and Tpit probably play a vital role in the MEKT1-mediated negative regulation of* Pomc* expression in AtT20 cells. Furthermore, although clinical trials of MEKT1 are needed to determine its drug efficacy in the future, it can be speculated that MEKT1 is much more effective than the previously recognized PPAR-*γ* agonists, rosiglitazone, and pioglitazone, for the suppression of* Pomc* expression/ACTH secretion from our* in vitro* research. Therefore, MEKT1 could be a novel therapeutic medication for the treatment of Cushing's disease.

## Figures and Tables

**Table 1:** Primer sequences for RT-qPCR.

Mouse Pomc	Forward	5′-CAGTGCCAGGACCTCACC-3′
	Reverse	5′-CAGCGAGAGGTCGAGTTTG-3′
Mouse PPAR-*γ*1	Forward	5′-TTCTGACAGGACTGTGTGACAG-3′
	Reverse	5′-ATAAGGTGGAGATGCAGGTTC-3′
Mouse PPAR-*α*	Forward	5′-AGACACGCAGACGGGTTG-3′
	Reverse	5′-GAGGATGCCACTCCCAGA-3′
Mouse PPAR-*β*	Forward	5′-TGGAGCTCGATGACAGTGAC-3′
	Reverse	5′- GTACTGGCTGTCAGGGTGGT-3′
Mouse Nur77	Forward	5′-GCACAGCTTGGGTGTTGATG-3′
	Reverse	5′-CAGACGTGACAGGCAGCTG-3′
Mouse Nurr1	Forward	5′-TCAGAGCCCACGTCGATT-3′
	Reverse	5′-TAGTCAGGGTTTGCCTGGAA-3′
Mouse NeuroD1	Forward	5′-ACGCAGAAGGCAAGGTGTCC-3′
	Reverse	5′-TTGGTCATGTTTCCACTTCC-3′
Mouse Tpit	Forward	5′-GCCAGCATGTGACCTACTCTCACT-3
	Reverse	5′-AGTCCAGCTGTCAGGTCCCGAGAA-3′
Mouse Pitx1	Forward	5′-CGGTGTGGACCAACCTCACTGAA-3′
	Reverse	5′-GAGTTGCACGTGTCCCGGTAGA-3′
Mouse NF*κ*B1	Forward	5′-GAAATTCCTGATCCAGACAAAAAC-3′
	Reverse	5′-ATCACTTCAATGGCCTCTGTGTAG-3′
Mouse NF*κ*B2	Forward	5′-CTGGTGGACACATACAGGAAGAC-3′
	Reverse	5′-ATAGGCACTGTCTTCTTTCACCTC-3′
Mouse Pttg	Forward	5′-CTGGGCACTGGTGTCAAG-3′
	Forward	5′-GCTGTTTTGGTTGGAGGGG-3′
Mouse GAPDH	Forward	5′-ACAGTCCATGCCATCACTGCC-3′
	Reverse	5′-GCCTGCTTCACCACCTTCTTG-3′

**Table 2:** Primer sequences for ChIP-qPCR.

Mouse NurRE	Forward	5′-ACACTGGGGAAATCTGATGC-3′
	Reverse	5′-CGGTGGTCAGGAGGAACTTA-3′
Mouse TpitRE	Forward	5′-GGCAGATGGACGCACATAGG-3′
	Reverse	5′-GCGCTGGTGGTTAGGAAGAA-3′
Mouse NBRE	Forward	5′-TTTCCAGGCAGATGTGCCTTGCGCT-3′
	Reverse	5′-CAGGGTTGGGTGGGTGAGCCTTGGA-3′

## Supplementary Materials

Supplementary Materials Figure S1: MEKT1-mediated effect of* PPAR-γ *mRNA expression in AtT20 cells in a dose-dependent manner. AtT20 cells were treated with MEKT1 (1 nM, 10 nM, 100 nM, 1 *μ*M, or 10 *μ*M) or 0.1% DMSO (vehicle control) for 24 hours. Data are expressed as percentages (100%) of control. Data represent mean ± SEM (*n* = 4). ^*∗∗*^*P* < 0.01 versus control. Figure S2: involvement of PPAR-*γ* in the MEKT1 effects on* Pomc* promoter activity. AtT20 cells transiently transfected with rPomc-luc, pRSV-*β*-gal, and siRNA (negative control; NC or PPAR-*γ*) for 48 hours were incubated in the presence of either MEKT1 (10 *μ*M) or 0.1% DMSO (control) for 24 hours, respectively. Results are expressed as percentages (100%) of control. Each point represents mean ± SEM (*n* = 4). NS stands for “not significant.” ^*∗∗*^*P* < 0.01 versus negative control of siRNA at 10 *μ*M MEKT1. Figure S3: MEKT1-mediated effects on the mRNA expression of Nur77,* Nurr1*, and* Tpit* in AtT20 cells are dose-dependent. (A) Dose-dependent effect of MEKT1 of* Nur77* mRNA expression. AtT20 cells were treated with MEKT1 (1 nM, 10 nM, 100 nM, 1 *μ*M, or 10 *μ*M) or 0.1% DMSO (vehicle control) for 24 hours. (B) Dose-dependent effect of MEKT1 on* Nurr1* mRNA expression. AtT20 cells were treated with MEKT1 (1 nM, 10 nM, 100 nM, 1 *μ*M, or 10 *μ*M) or 0.1% DMSO (vehicle control) for 24 hours. (C) Dose-dependent effect of MEKT1 on* Tpit *mRNA expression. AtT20 cells were treated with MEKT1 (1 nM, 10 nM, 100 nM, 1 *μ*M, or 10 *μ*M) or 0.1% DMSO (vehicle control) for 24 hours. Each point represents mean ± SEM (*n* = 4). Data are presented as percentages of control (100%). ^*∗*^*P* < 0.05, ^*∗∗*^*P* < 0.01, and ^*∗∗∗*^*P* < 0.001 versus control. Figure S4: effects of Nur77, Tpit, and Nurr1 overexpression on MEKT1-mediated effect of promoter activity of* Pomc*. AtT20 cells were transiently transfected with pcDNA3 and Nur77 overexpression plasmid in (A), Tpit overexpression plasmid in (B), and Nurr1 overexpression plasmid in (C) and 135 ng of rPomc-Luc and 65 ng of pRSV-*β*-gal were incubated either in the presence of MEKT1 at 10 *μ*M or DMSO at 0.1% (control) for 24 hours before the luciferase assay. Each overexpression plasmid volume was maintained at 300 ng adding pcDNA3 empty vector. Results are expressed as percentages of each control (100%). Data represent mean ± SEM (*n* = 4). NS means “not significant.” ^*∗*^*P* < 0.05 versus control.
