# Supplementary material for: Inhibitory Effects of a Novel PPAR-γ Agonist MEKT1 on Pomc Expression/ACTH Secretion in AtT20 Cells
Source: PPAR Res. 2018 Apr 23;2018:5346272. doi: 10.1155/2018/5346272 (PMC5937427; doi:10.1155/2018/5346272)
Supplement: Supplementary Materials — Figure S1: MEKT1-mediated effect of PPAR-γ mRNA expression in AtT20 cells in a dose-dependent manner. AtT20 cells were treated with MEKT1 (1 nM, 10 nM, 100 nM, 1 μM, or 10 μM) or 0.1% DMSO (vehicle control) for 24 hours. Data are expressed as percentages (100%) of control. Data represent mean ± SEM (n = 4). ∗∗P < 0.01 versus control. Figure S2: involvement of PPAR-γ in the MEKT1 effects on Pomc promoter activity. AtT20 cells transiently transfected with rPomc-luc, pRSV-β-gal, and siRNA (negative control; NC or PPAR-γ) for 48 hours were incubated in the presence of either MEKT1 (10 μM) or 0.1% DMSO (control) for 24 hours, respectively. Results are expressed as percentages (100%) of control. Each point represents mean ± SEM (n = 4). NS stands for “not significant.” ∗∗P < 0.01 versus negative control of siRNA at 10 μM MEKT1. Figure S3: MEKT1-mediated effects on the mRNA expression of Nur77, Nurr1, and Tpit in AtT20 cells are dose-dependent. (A) Dose-dependent effect of MEKT1 of Nur77 mRNA expression. AtT20 cells were treated with MEKT1 (1 nM, 10 nM, 100 nM, 1 μM, or 10 μM) or 0.1% DMSO (vehicle control) for 24 hours. (B) Dose-dependent effect of MEKT1 on Nurr1 mRNA expression. AtT20 cells were treated with MEKT1 (1 nM, 10 nM, 100 nM, 1 μM, or 10 μM) or 0.1% DMSO (vehicle control) for 24 hours. (C) Dose-dependent effect of MEKT1 on Tpit mRNA expression. AtT20 cells were treated with MEKT1 (1 nM, 10 nM, 100 nM, 1 μM, or 10 μM) or 0.1% DMSO (vehicle control) for 24 hours. Each point represents mean ± SEM (n = 4). Data are presented as percentages of control (100%). ∗P < 0.05, ∗∗P < 0.01, and ∗∗∗P < 0.001 versus control. Figure S4: effects of Nur77, Tpit, and Nurr1 overexpression on MEKT1-mediated effect of promoter activity of Pomc. AtT20 cells were transiently transfected with pcDNA3 and Nur77 overexpression plasmid in (A), Tpit overexpression plasmid in (B), and Nurr1 overexpression plasmid in (C) and 135 ng of rPomc-Luc and 65 ng of pRSV-β-gal were incubated either in [file 5346272.f1.pdf]

Inhibitory effects of a novel PPAR-γ agonist MEKT1 on *Pomc* expression/ACTH secretion in AtT20 cells

Figure Legends of Supplimentary

Figure S1

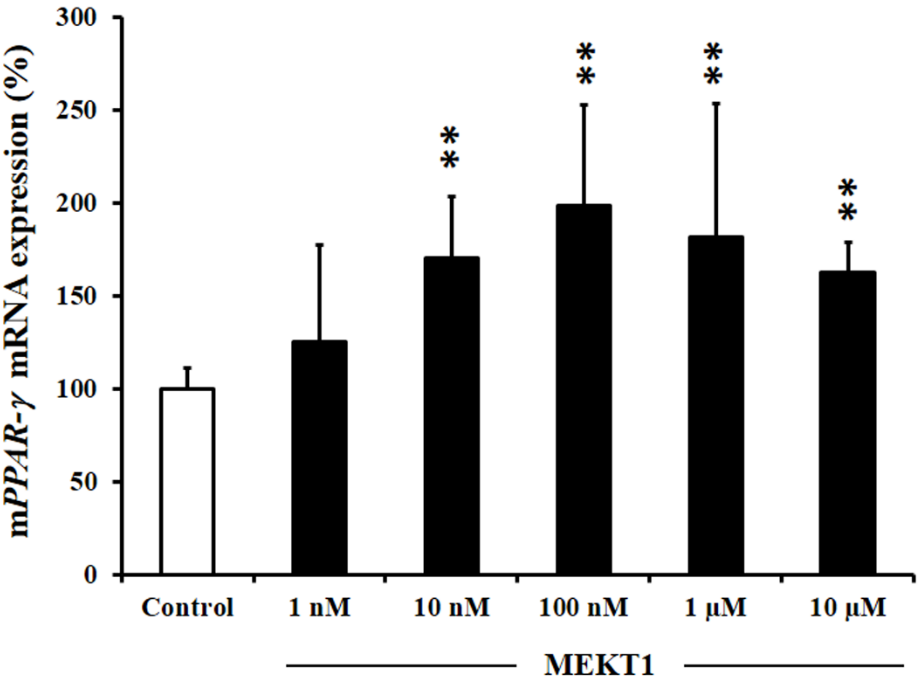

Figure S1. MEKT1-mediated effect of *PPAR-γ* mRNA expression in AtT20 cells in a dose-dependent manner. AtT20 cells were treated with MEKT1 (1 nM, 10 nM, 100 nM, 1 µM, or 10 µM) or 0.1% DMSO (vehicle control) for 24 hours. Data are expressed as percentages (100%) of control. Data represent mean ± SEM (n=4). **P<0.01 vs control.

Figure S2

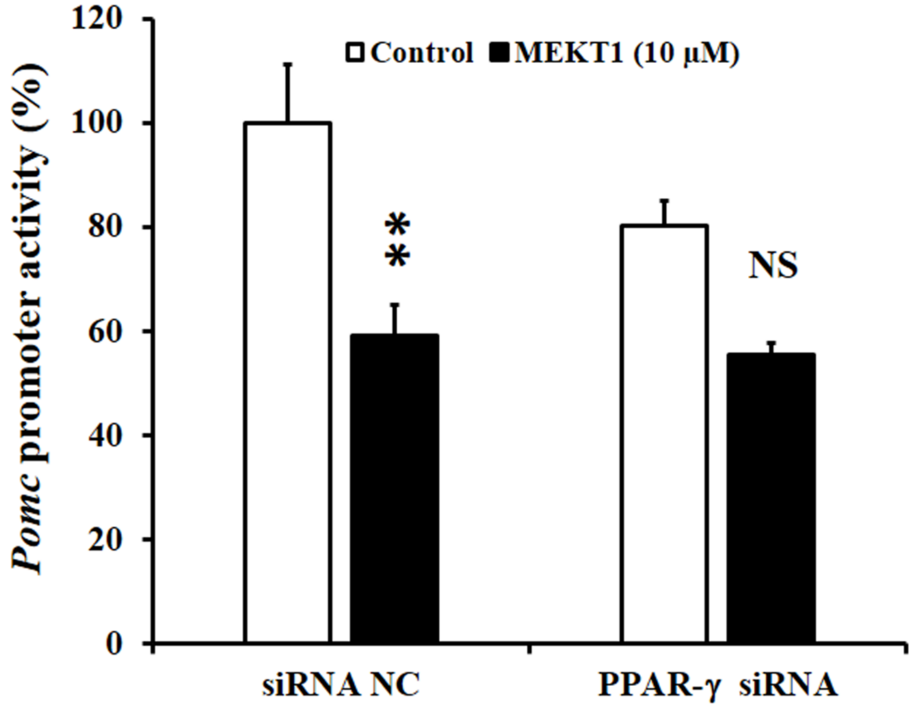

Figure S2. **Involvement of PPAR-γ in the MEKT1 effects on *Pomc* promoter activity.** AtT20 cells transiently transfected with rPomc-luc, pRSV-β-gal, and siRNA (negative control; NC or PPAR-γ) for 48 hours were incubated in the presence of either MEKT1 (10 µM) or 0.1% DMSO (control) for 24 hours, respectively. Results are expressed as percentages (100 %) of control. Each point represents mean ± SEM (n = 4). NS stands for "not significant." **P<0.01 vs negative control of siRNA at 10 µM MEKT1.

.

Figure S3

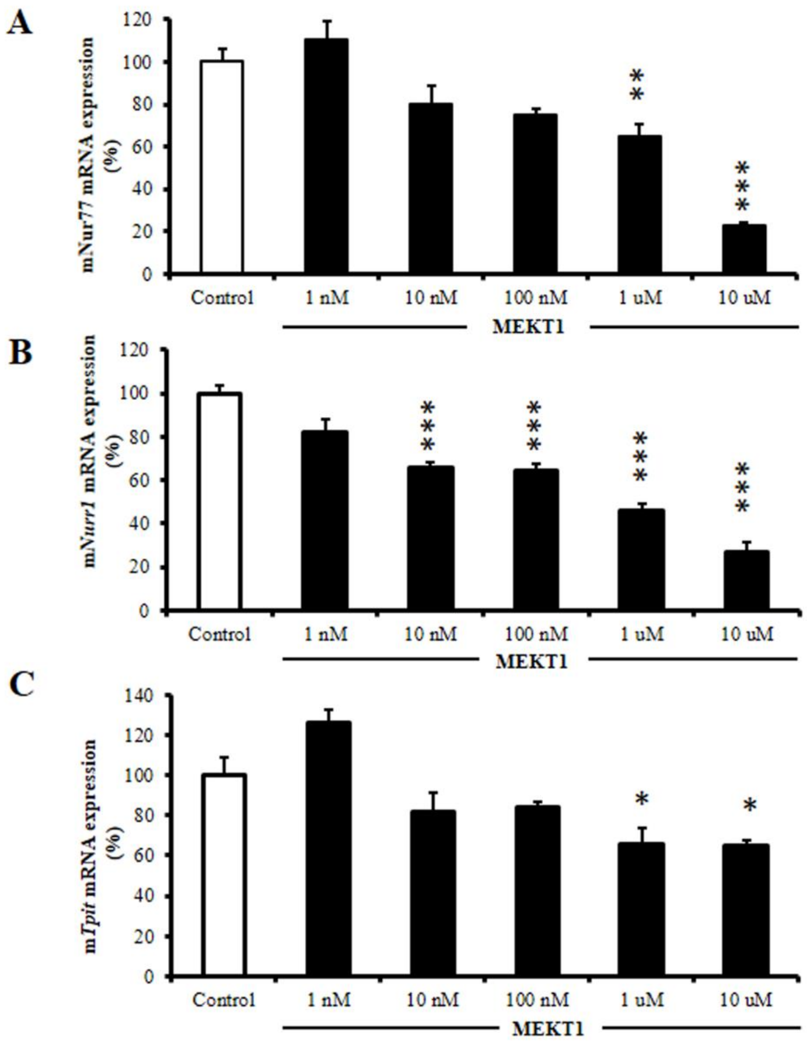

Figure S3. MEKT1-mediated effects on the mRNA expression of Nur77*,* *Nurr1*, and *Tpit* in AtT20 cells are dose-dependent.

(A) Dose-dependent effect of MEKT1 of *Nur77* mRNA expression. AtT20 cells were treated with MEKT1 (1 nM, 10 nM, 100 nM, 1 µM, or 10 µM) or 0.1% DMSO (vehicle control) for 24 hours. (B) Dose-dependent effect of MEKT1 on *Nurr1* mRNA expression. AtT20 cells were treated with MEKT1 (1 nM, 10 nM, 100 nM, 1 µM, or 10 µM) or 0.1% DMSO (vehicle control) for 24 hours. (C) Dose-dependent effect of MEKT1 on *Tpit* mRNA expression. AtT20 cells were treated with MEKT1 (1 nM, 10 nM, 100 nM, 1 µM, or 10 µM) or 0.1% DMSO (vehicle control) for 24 hours. Each point represents mean ± SEM (n=4). Data are presented as percentages of control (100%). *P<0.05, **P<0.01, ***P<0.001 vs control.

Figure S4

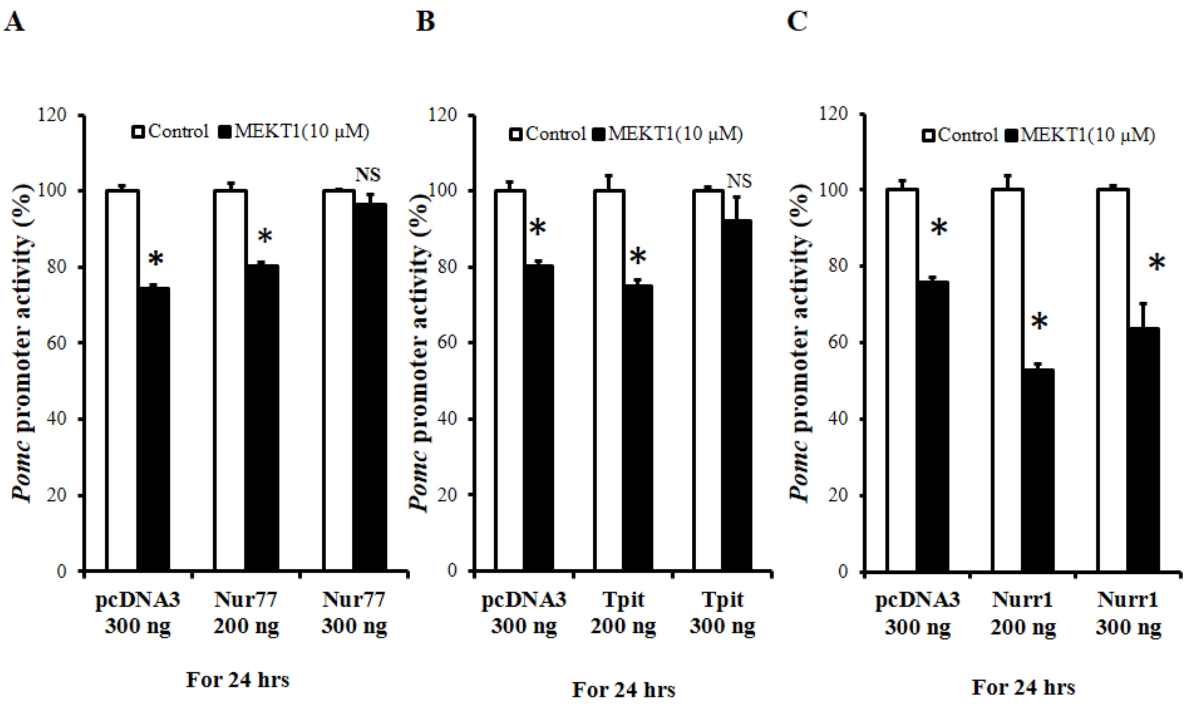

Figure S4. Effects of Nur77, Tpit, and Nurr1 overexpression on MEKT1-mediated effect of promoter activity of *Pomc*. AtT20 cells were transiently transfected with pcDNA3 and Nur77 overexpression plasmid in (A), Tpit overexpression plasmid in (B), and Nurr1 overexpression plasmid in (C) and 135 ng of rPomc-Luc, and 65 ng of pRSV-β-gal were incubated either in the presence of MEKT1 at 10 µM or DMSO at 0.1 % (control) for 24 hours before the luciferase assay. Each overexpression plasmid volume was maintained to 300 ng adding pcDNA3 empty vector. Results are expressed as percentages of each control (100%). Data represent mean ± SEM (n=4). NS means "not significant." *P<0.05 vs control.
